# Evaluation of IgY Antibody as a Polyspecific Coombs-Reagent

**Published:** 2017

**Authors:** Esteban Justo Gutiérrez Calzado, Marlene Toledano Heredia, Jeorge Fernadez Duharte, Amir-Hassan Zarnani

**Affiliations:** 1. Laboratory of Antibodies and Experimental Biomodels, Center of Molecular Immunology, Santiago de Cuba, Cuba; 2. Department of Immunology, Reproductive Biotechnology Research Center, Avicenna Research Institute, ACECR, Tehran, Iran

**Keywords:** Chicken, Egg yolk antibody, Immunization, Immunoglobulins

## Abstract

**Background:**

During the last twenty years, the extraction of specific egg yolk (IgY) antibodies from the immunized chickens has been accepted as a useful alternative to the immunization of mammals. The aim of the present study was immunizing the chickens with Human Umbilical Cord Serum (HUCS) and the extraction of specific anti-human globulins (IgG, C3b, and C3d) antibodies from egg yolk in order to obtain polyspecific Coombs reagent.

**Methods:**

The novelty of this work was the achievement of a polyclonal reagent through a very cheap alternative method in accordance with all ethical regulations required for obtaining it. Three Leghorn hens (21 weeks old) were immunized four times for a period of 66 days with 20uL of HUCS mixed with PBS/FCA or FIA each time. The extraction of IgY antibodies was performed according to the method of lipid precipitation of yolk and using water soluble fraction as the reagent material. The resulting IgY antibody was characterized by SDS-PAGE and immunoelectrophoresis and tested for the presence of hetero-agglutinins by means of direct agglutination using human erythrocytes of all blood groups treated with 0.1% papain and for indirect Coombs-test to evaluate its specificity to fractions (C3b, C3d, C4d) of human complement and human IgG, respectively.

**Results:**

Our findings show, that, the reagent obtained contains IgY and other 3 proteins (SDS-PAGE), and reacts specifically with plasma proteins, that migrate in β and ϒ regions. In immunoelectrophoresis, in addition, there is the presence of low hetero-agglutinins levels in IgY-preparation (3 lots), and the possibility to produce high amount (more than 500 *ml/egg*) of polyspecific Coombs-reagent in chickens is also discussed.

**Conclusion:**

IgY-preparation (3 lots), and the possibility to produce high amount (more than 500 *ml/egg*) of polyspecific Coombs-reagent in chickens with the originality to achieve a polyclonal reagent through a very cheap alternative method in accordance with all ethical regulations required for obtaining it, was also discussed.

## Introduction

Human antiglobulin or Coombs-Test represents the most important artificial agglutination in immunohematology. By immunological procedure, the Coombs’ reaction reveals the presence of non agglutinant antibodies in erythrocyte membrane. The Coombs’-Test is an immunological procedure consisting of antibodies against human antibodies. These antibodies can recognize each type of human Igs, and red blood cells and maintain linkage through specific antibodies to membrane antigens. There are polyspecific and monospecific Coombs’ sera. Polyspecific Coombs’ sera contain antibodies against Fc fractions of Igs and antibodies against human complement fractions (C3b and C3d, C4d) that can be presented by sensibilization of erythrocyte membrane. Monospecífic Coombs’ sera contain antibodies against a specific kind of Ig (IgG, IgM or IgA) or against complement fractions (C3b or C3d, C4d). The CoombsTest can be performed in two ways: direct and indirect Coombs ^1–3^.

Since the first report in 1945 ^4^, the Coombs-serum has been produced by immunizing mammals using whole serum from apparently healthy blood donors, or using globulin fractions obtained from whole blood, and, this procedure is still practiced ^4,5^. In addition, there are reports on the use of colostrum as Coombsreagent by immunizing cows with human whole blood. Since a few years ago, monoclonal antibodies with respective specificities are obtainable (*e.g*. DAKO, Denmark; Spinreact, Spain). However, currently, the data is unavailable with respect to standardization or validation of the production of Coombs-serum. It concerns the aspects of immunization protocols, the species of mammals chosen, use of adjuvants, *etc* ^4–6^.

In the last twenty years, the IgY technology has attracted the interests of scientific community and enterprises due to its non-invasive antibody production, and as a potential alternative to antibody production in mammals ^7–10^. The chicken sourced IgY antibodies present various significant advantages over mammalian IgG antibodies. Chickens produce an enormous amount of IgY antibodies in egg yolk in contrast to rabbits (during a month at least 1500 *mg* total IgY can be sampled from one laying hen compared to appr. 200 *mg* total IgG from a rabbit) ^11^. Antibody sampling from hens is non-invasive as it is extracted from egg yolk, and eggs are collected on daily basis for long period of time. Due to the phylogenetic distance between birds and mammals, the immune system of chickens responds well to highly conserved antigens which do not elicit strong immune response in mammals ^10,12,13^.

In the last years, IgY-technology is used in many fields of research. For instance, it is used in diagnosis of various bacterial or viral diseases in human and veterinary medicine, in detection of prohibited or harmful drug residuals in food or foodstuff, in qualitative and quantitative measurement of numerous bioactive proteins and peptides, and, most recently, in studies for detection and quantitation of biomarkers for diagnostics of several diseases and physiological conditions as inflammation, thalassemia, atherosclerosis, bovine tuberculosis and regeneration of skeletal muscle ^14–18^ and for diagnosis and prognosis of several types of cancer ^19–24^.

There are several studies relating to the production of monospecific Coombs-reagent from egg yolk IgY with specificity against human IgG ^25^, but it is possible to improve bio-model (concerning in particular, changes in immunization protocol, to increase the ab titer), and on the other hand, the use of red blood cells treated with proteolytic enzymes to improve the sensibility of indirect Coombs-test since it is very important to evaluate international recommendations for its production.

It is not recommended to obtain polyspecific Coombs-reagent by immunizing animals with human total serum, because, in this way, it is possible to avoid the presence of antibodies against C4 fraction of human complement to eliminate cross-reaction with antigens from Chido/Rodgers red blood cell group ^1^. The elimination of this cross-reaction could decrease the general levels of hetero-agglutinins present in the obtained re-agent.

The fact of the presence of very low quantities of C4 complement fractions in chosen human umbilical cord sera in comparison with high level of C3 justifies the main goal of our present study.

This work consisted of the use of raw material (human umbilical cord sera) for immunizing chickens, to evaluate the possible production of polyspecific Coombs-reagent, with the originality to achieve a polyclonal reagent through a very cheap alternative method in accordance with all ethical regulations required for obtaining it.

## Materials and Methods

### Antigen for immunization

Blood samples from umbilical cord were taken according to Ethical Regulation No.1–99, Annex No.1–4 signed in Drugs Quality Control Center (CECMED) in Cuba according to specificity of quality of blood from donors.

Concentrations of globulin were determined to thirteen samples of human umbilical cord sera from newborns donors by Turbidimetry test (SCIENTIFICA TECNOLOGIE BIOMEDICHE, Italy); three of them were used as immunogenic material.

### Animals

Three chickens (White Leghorn, 21 weeks old) were obtained from a commercial breeder (Cuban Centre for the Production of Laboratory Animals, CENPALAB, Havana, Cuba) and were kept individually in cages (128×65×80 *cm*), water and food were provided (special diet CM 005 Al y Co, CENPALAB, Havana, Cuba) *ad libitum,* according to general ethical regulations in Cuba, and to a great extent to the recommendation of the 21^st^ ECVAM workshop on IgY-technology^11^.

The immunization of animals was done via left and right breast with 20 *uL* (approx. 800 *ug* of C3, 5 *ug* of C4 and 300 *ug* of human IgG) from human umbilical cord serum, with frequency of 0, 15, 36, 66 days with volume 1.0 *mL* of phosphate-buffered saline and PBS was added to umbilical cord serum to complete 500 *uL* and for mixing with Freund’s Complete Adjuvant (FCA, first immunization, SIGMA, Germany) or Freund’s Incomplete Adjuvant (FIA, booster-immunizations, SIGMA, Germany) in a ratio of v:v (umbilical cord serum-PBS:FCA/FIA).

### Collection of eggs and IgY extraction

The eggs were collected between days 73 to 80 after first immunization, and subsequently stored at 4°*C* for further processing. The IgY antibodies of 15 collected eggs were extracted according to the method previously described ^8^. Egg yolk was mixed with distilled water (pH=5.0 by adding HCL) in a ratio of 1:9. The solution was centrifuged (2.500 *rpm*, 4°*C*, 30 *min*) with refrigerated centrifuge (Medifriger, Selecta, Abrera, Spain) and NaCL (0.15 *M*) was added to the supernatant and the pH was adjusted to 7.2 with Tris salt. To avoid bacterial contamination, sodium azide (NaN_3_) was added in a concentration of 0.1%. The IgY-preparations (later on indicated as IgY-reagent) were stored at 4°*C* (3 lots: each for 5 eggs/hen).

The principal advantage of IgY technology is to provide a noninvasive method for the production of antibodies consistent with the principle of the 3RS. This, coupled with the enormous amount of antibodies that provide an egg yolk, and knowledge already established in the literature, shows no significant differences in the levels of antibodies in serum and yolk, manifesting sufficient reasons for not doing monitoring of antibody titers in serum of immunized animals ^7,11,25^.

### Determination of total protein concentration

The total protein concentration was measured in supernatant obtained by protocol using Lowry method, ^26^ with bovine seroalbumin as protein standard (Sigma Co. Ltd). Absorbance was read at 660 *nm* in Spectrophotometer Ultrospec 1000 (Pharmacia. LKB).

### Total IgY concentration

A Single Radial Immunodifussion Method or Mancini Test ^27^ was used for measuring the total IgY content in egg yolk supernatant (IgY-Preparation). The test included a commercial rabbit anti IgY antiserum and standard IgY preparation (Sigma Co. Ltd).

### Sodium Dodecyl Sulfate Polyacrylamide Gel Electrophoresis (SDS-PAGE)

According to, Laemmli method, ^28^ a 5% stacking gel, and 12.5% separating gel, respectively, were used. The electrophoretic run was made with the help of Power Supply EPS-600 (Pharmacia), and no reducing conditions.

### Immunological specificity

An immunoelectrophoretical test ^27^ was carried out to show the reactivity of IgY-preparation. A total human serum and a commercial preparation of human IgG (IMEFA, Cuba) in 1% solidified agar plate were used to test the specific reactivity of IgY-preparation.

### Testing for the presence of hetero-agglutinins

Three lots of IgY-preparation were tested on the existence of hetero-agglutinins by means of direct agglutination, using erythrocytes of all blood groups treated with 0.1% papain (100 *uL* of IgY preparation was added to 100 *uL* of an erythrocyte preparation 2% in 0.15 *moL/L* NaCL-solution of each human blood group) (ABO), mixed gently, and kept for 1 *hr* at room temperature. Thereafter, the mixture was centrifuged for 1 *min* (1000 *r/min*) and, subsequently, the presence of agglutination was assessed.

### Testing of specific reactivity of IgY -preparation

Furthermore, the reactivity of the obtained IgY-re-agent (three lots) was tested using erythrocytes coated with commercial human anti-D IgG antibody (DAKO, Denmark) for the determination of anti-human IgG titers. For the determination of antibody specificity, the three lots of IgY-preparation were tested by a hemagglutination procedure (Indirect Coombs-Test), using erythrocytes coated with different fractions of human complement (C3b, C3d, and C4d).

## Results

**Figure 1.**
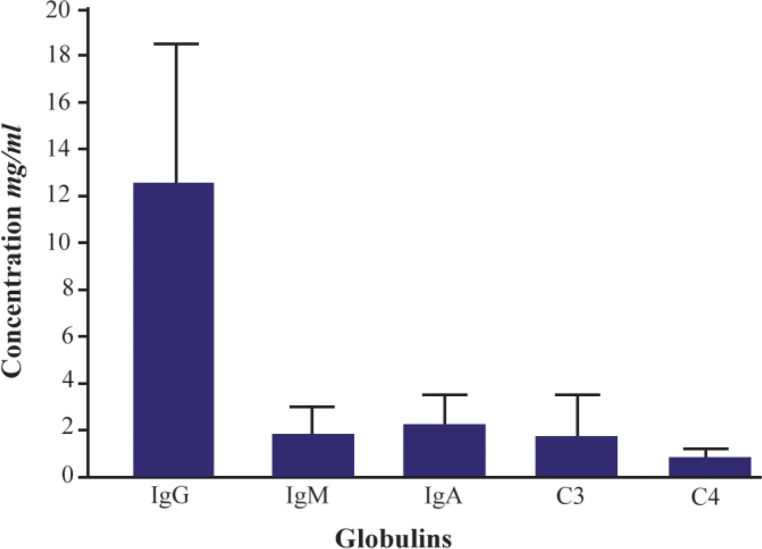
Standard concentrations of globulins in human normal serum.

**Figure 2.**
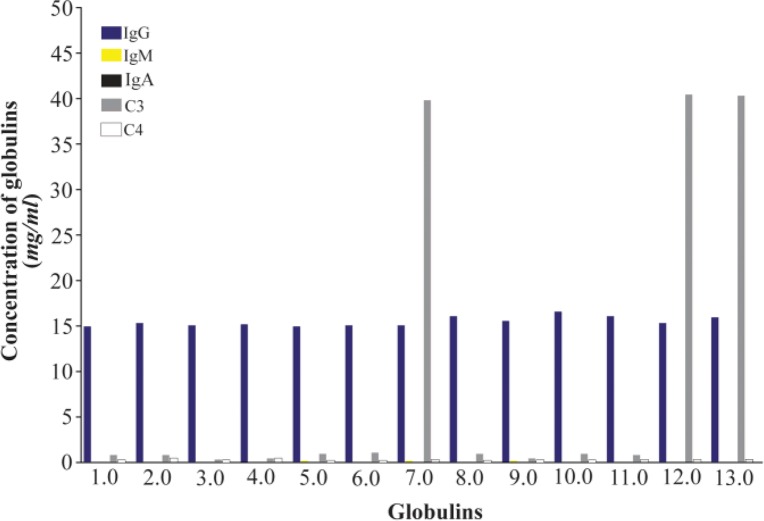
Concentrations of globulins in thirteen samples of umbilical cord sera. See no presence of IgA and IgM in any sample. There is very high C3 (complement fraction) concentration in three of them (in red).

Standard concentrations of globulins in human normal serum are presented in figure 1 and the ranges of values for human complement fractions C3 and C4 are between 0.1 to 3.5 *mg/mL* (1.8 *mg/mL* as mean value approx.) and 0.5 to 1.2 (0.85 as mean value approx.), respectively and the mean values reported for C3 and C4 in umbilical cord sera were 0.8 *mg/mL* and 0.12 *mg/mL*, respectively. There was no evidence for presence of IgA and IgM in umbilical cord sera because these molecules do not cross the placenta (Figure 2).

Taking in mind that most of 90% of diagnostic capacity of Coombs-Test can be achieved with two specificities of Coombs- reagent (anti human IgG and C3), the search for antibodies response against these two components is the fundamental purpose in any attempt to achieve this goal.

**Figure 3.**
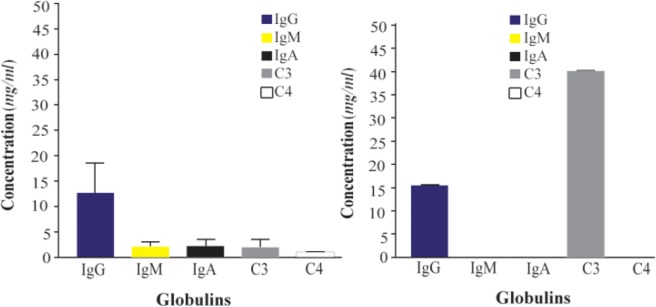
Comparison of concentrations for globulins between human normal sera (left) and chosen umbilical cord sera samples (right).

In order to guarantee appropriate raw material for immunizations, umbilical cord sera were selected. In our current study, concentrations of plasmatic globulins were measured (IgG, IgA, IgM, C3 and C4 fractions of human complement). It is known that antibodies against fraction C4 produce cross-reaction with Chido-Rodgers blood group antigen ^1,2^, and trying to obtain IgY antibody with low levels, or no response against fraction C4 of human complement, three from thirteen umbilical cord sera with very high levels of C3 were chosen (Figures 2 and 3), in this case, expecting low levels of hetero-agglutinins in our IgY reagent.

**Figure 4.**
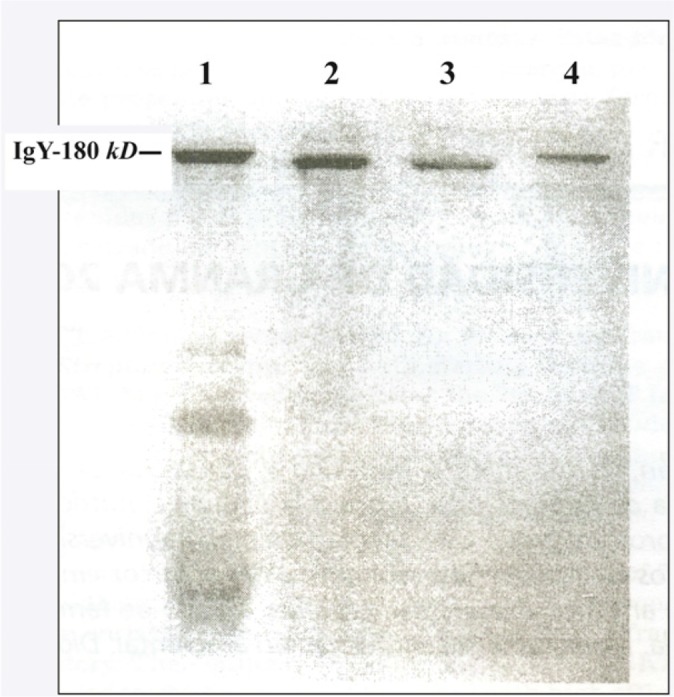
SDS-PAGE (no reducing conditions). 1. Specific IgY-Preparation against chosen human umbilical cord sera by WSF method (50 *μl* of preparation, aprox. 50 *μg*). 2. Standard of human IgG (20 *μg*). 3. Standard of mouse monoclonal IgG (10 *μg*). 4. Standard of chicken IgY from Sigma (10 *μg*). Compare lanes 1 and 4. The position of upper band in lane 1 correspond to band of pure IgY (180 *kDa* approx.) in lane 4. See the bands in lanes 2 and 3 underneath 180Kd that correspond to mammals IgG monomers (150 *kDa* approx).

SDS-PAGE (Figure 4) shows the characteristics of reagent (IgY-preparation) later tested in hemagglutination assays. The reagent obtained contained four bands of proteins. Similar results have been obtained by Fichtali ^9^ which describes IgY, and chicken sero albumina among others. Total IgY represents approximately 23% in comparison to total protein concentration (Table 1). This agrees with the data published by Fichtali ^9^ *et al* who described that content of IgY in egg yolk supernatant is approx. 20% in comparison with other contaminant proteins. This finding was achieved for the author for densitometry studies of egg yolk supernatant in SDS-PAGE.

**Table 1.** Main parameters in the process of IgY-preparation

**Main parameters**	**Mean Value**
**Number of processed eggs**	15
**Weight of egg (g)**	60.9±1.94
**Volume of yolk (*ml*)**	14.0±1.18
Volume of egg yolk supernatant (*ml*)^*^	111.0±2.5
**Total protein concentration (Lowry) (*mg*)**	498.92±15.11
Total IgY concentration (Mancini) (*mg*)^**^	114.97±7.51

* Represents the total volume of IgY preparation after processing of each yolk by Water Soluble Fraction Method (WSF).

** Represents 23% of total protein concentration.

The presence of chicken albumin could be important because, normally, bovine albumin is necessary as an additive in Coombs-reagent to improve the immunological reaction, and to avoid incorrect results ^29,30^. The IgY-preparation could eliminate this step with corresponding saving.

**Figure 5.**
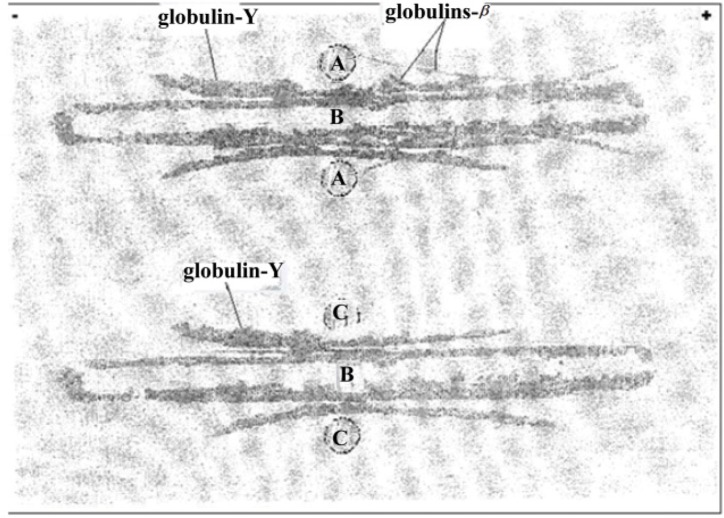
Immunoelectrophoresis. (A) Total human serum; (B) IgY-Preparation against chosen umbilical cord sera; (C) Commercial human IgG. Globulins-β (Globulins Beta) represents two specific arcs of precipitation toward β region (C3, C4) of migration of total plasma globulins and globulin-Y(Globulins Gamma) represent specific arc of precipitation that migrates toward Y region (human IgG) of total plasma globulins (Top part of the figure). Lower of the figure represents specific arc of precipitation toward human IgG to compare sense of migration in immunoelectro-phoresis.

In fact, immunoprecipitation’s arcs, listed in immunoelectrophoresis (Figure 5), correspond to components belonging to ϒ and β regions from immunoelectrophoretical running of human globulins (IgG and complement fractions) and it indicates that reagent obtained is ready for testing as polyspecific Coombs serum.

Each egg yolk can supply more than 100 *mL* (111 *mL* approx.) of material rich in IgY with more than 100 *mg* of total IgY (114 *mg* approx.) (Table 1). This fact demonstrates the enormous amount of reagent which gives us this preparation.

**Figure 6.**
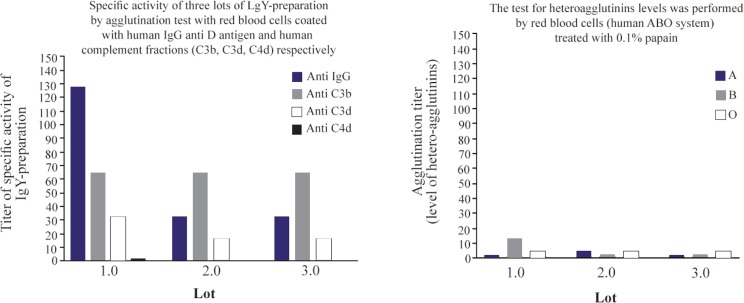
Specific activity of original IgY-preparation and the presence of hetero-agglutinins levels.

Figure 6 shows the titer of egg yolk antibodies against human IgG, complement fractions C3b, C3d, and C4d and the presence of hetero-agglutinins. The fact that specific antibody titers in lot 1 are greater than in lots 2 and 3 responds to these, departed from different animals, which by its own inherent particularities, antibody responses have certain differences. The use of red blood cells treated with proteolytic enzymes to improve the sensibility of indirect Coombs–Test is recommended by international regulations to evaluate liberation of lots of reagent, specifically, the use of erythrocytes treated with 0.1 % papain ^1,31^.

Treatment of red blood cells with proteolytic enzymes is very important in enzymatic techniques of artificial agglutination. Trypsin, papain, bromeline and ficin are the most important proteolytic enzymes used in these cases.

These enzymes have the capacity to expose peptidic fragments of glycoproteins to erythrocyte membrane. These fragments contain sialic acid molecules (electro negatives) that decrease negative charge in red blood cells. As the consequence of decreasing the electronegativity in red blood cells, shields of positive ions (Na^+^), attracted near erythrocyte membrane, also decrease and value of electric potential difference (zeta potential) between the shields and suspending medium in equilibrium (neutral), also decreases. For lower values of zeta potential (Z), suspensions of red blood cells are more agglutinable. This agrees with Pollack’s electrostatic model where Z is directly proportional to electronegativity of red blood cells. For example, red blood cells treated with cited proteolytic enzymes can be agglutinated in salt solution by class IgG anti Rh-D ab (non agglutinant) ^1,3^.

Although these results show that IgY reagent obtained in our study manifests the presence of hetero-agglutinins, their levels are very low in comparison with specific activity for good performance of reagent (Figure 6). On the other hand, when we compare similar traditional reagents obtained in rabbits in our lab for many years, the levels of hetero-agglutinins in these mammals are very higher (data no shown).

The international regulations for the acceptance of polyspecific Coombs-reagent assigned titers are 8, 4 and 2 for activities of anti IgG, C3b, C3d respectively, negative agglutination to C4 component, and, no presence of hetero-agglutinins when you put reagent in front of ABO panel of hematies treated with 0.1% papain ^31^. Our current study shows better results in comparison to traditional reagents in mammals (personal experience not shown) where IgG antibodies recognize fraction C4d. Two lots of IgY antibodies presented no reaction to C4d, maybe because of the procedure that we used, and lots of polyspecific Coombsreagents absolutely absent of anti C4 reaction can be achieved, eliminating last tedious steps to eliminate hetero-agglutinins in general.

**Figure 7.**
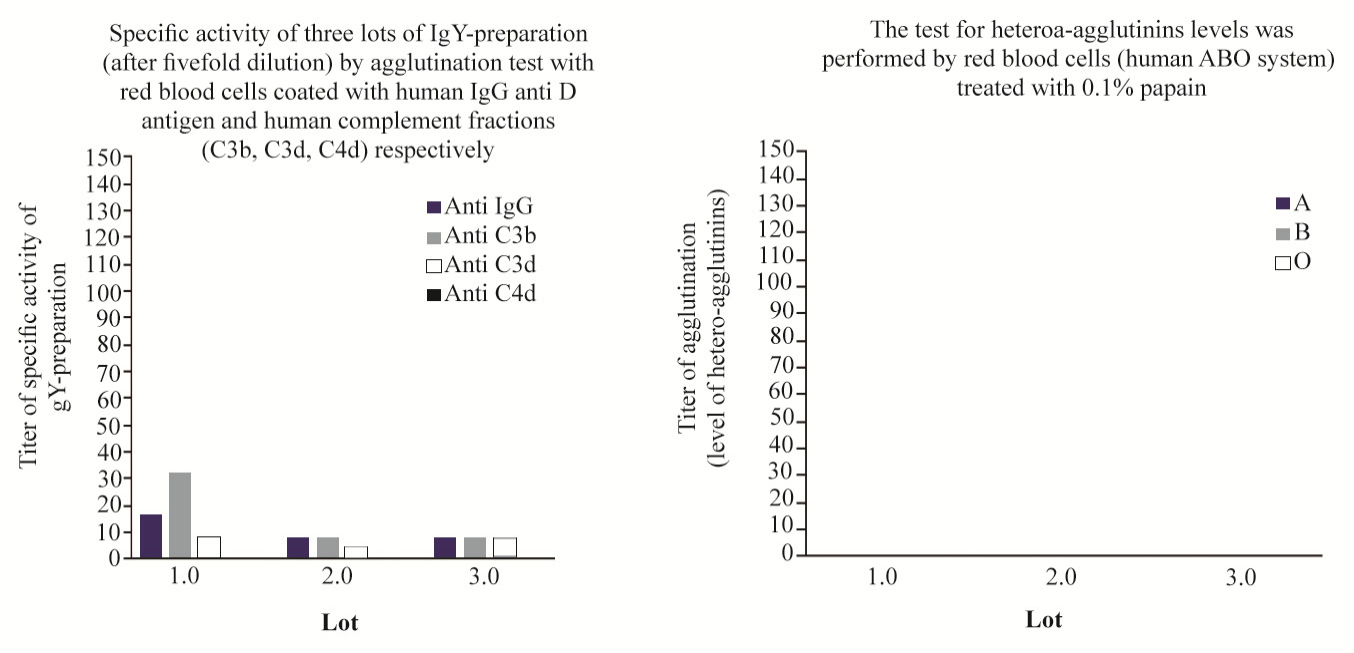
Specific activity of IgY-preparation after fivefold dilution of reagent (left) and demonstration of no presence of hetero-agglutinins in it (right).

Figure 6 shows that the reagent obtained in our work, in principle, fails this regulation ^31^. The three lots of IgY underwent fivefold dilution and were submitted again to test for specific activity and the presence of hetero-agglutinins. The results shown in figure 7 demonstrate that, with the single procedure of dilution, lots of IgY reagents obtained meet the international recommendations for the production of polyspecific Coombs-reagent, with the advantage to avoid several tedious and expensive steps, as chromatography methods, and others, to eliminate the presence of agglutinins that cause cross reaction and false positive performance of direct and indirect Coombs-Test. In addition, the stability of reagent was maintained for 6 months (data no shown). All assays were performed, in parallel, with polyspecific antiglobulin reagent from commercial source (Spinreact, Spain) as control. In all cases, specific activity anti IgG and anti C3 was 1: 128 and 1:64, respectively. No heteroaglutinin was present in any case of positive control reagent.

## Discussion

Despite the presence of drawbacks in original IgY reagent (before dilution) and since it is not recommended to try to obtain polyspecific Coombs-reagent, immunizing animals with human total serum, is possible to avoid the presence of antibodies against C4 fraction of human complement to eliminate cross-reaction with antigens from Chido/Rodgers red blood cell group ^1^ in order to decrease the hetero-agglutinins present in the reagent obtained. The present study demonstrated the fact that in using chosen umbilical cord sera as raw material (high level of C3) to immunize chickens, it is possible to achieve an IgY-preparation with the presence of very low activity of anti C4 complement fraction, and hetero-aglutinins let us achieve a preparation of IgY antibody from egg yolk by single dilution, that can meet the international recommendations for this diagnostic reagent.

The better results obtained in this work, in conjunction to the non-invasive IgY antibody sampling, and the enormous amount of specific ab obtainable [one egg can supply more than hundred mL of original reagent (Table I)] and the fivefold dilution, more than 500 *mL*, that represents approx. five thousands determinations in immunohematology tests (100 *uL* each one), besides low quantity of hetero-agglutinins, in general, in comparison to similar ones obtained in mammals, justify the introduction of this innovative technology that will let us evaluate it in direct and indirect Coombs-test. The results, can demonstrate, in the first case, *in vivo* sensibilization of red blood cells by antibodies or human complement fraction for diagnosis of hemolytic anemia of newborn’s disease, autoimmune hemolytic anemia, and, hemolysis induced by drugs and post-transfusional hemolytic reactions, and in the second case, execution of several tests like investigation and identification of antibodies anti red blood cells, tests of pre-transfusional compatibility, and determination of erythrocyte antigens that can not be shown by direct agglutination (eg: RhD weak variants, Duffy, Kidd, Kell antigens, *etc.*) ^1,3,31^.

In addition, the reagent obtained is expected for further commercialization based on its presented effectiveness and competitive cost.

## Conclusion

The present study demonstrates the fact that using chosen umbilical cord sera as raw material (high level of C3) for immunizations of chickens to achieve an IgY-preparation with the presence of very low levels of activity of anti C4 of complement fraction and heteroaglutinins provided the chance to achieve a preparation of IgY antibody from egg yolk by single dilution, that can meet the international recommendations for this diagnostic reagent.

Taking into consideration that enterprises engaged in the production of reagents for immunohematology uses (Spinreact, DAKO, *etc*.) produce Coombs-reagent with monospecific activity, mainly *via* bioreactors, a debate over achieving an alternative method, less tedious, and, less expensive for the production of anti human globulin reagent was presented. This proposed method is expected to be effective and cost competitive in commercialization around the world.
